# Evidence-based pregnancy testing in clinical trials: Recommendations from a multi-stakeholder development process

**DOI:** 10.1371/journal.pone.0202474

**Published:** 2018-09-12

**Authors:** Jessica E. Morse, Sara B. Calvert, Claire Jurkowski, Melissa Tassinari, Catherine A. Sewell, Evan R. Myers

**Affiliations:** 1 Department of Obstetrics and Gynecology, University of North Carolina School of Medicine, Chapel Hill, North Carolina, United States of America; 2 Clinical Trials Transformation Initiative, Duke University Medical Center, Durham, North Carolina, United States of America; 3 Global Pharmacovigilance and Epidemiology, Bristol-Myers Squibb, Hopewell, New Jersey, United States of America; 4 Center for Drug Evaluation and Research, US Food and Drug Administration, Silver Spring, Maryland, United States of America; 5 Department of Obstetrics & Gynecology and Duke Clinical Research Institute, Duke University Medical Center, Durham, North Carolina, United States of America; Hvidovre Hospital, DENMARK

## Abstract

Most clinical trials exclude pregnant women in order to avoid the possibility of adverse embryonic and/or fetal effects. Currently, there are no evidence-based guidelines regarding appropriate methods for identifying early pregnancy among research subjects. This lack of guidance results in wide variation in pregnancy testing plans, leading to the potential for inadequate protection against embryonic or fetal exposure in some cases and unnecessary burdens on research participants in others, as well as inefficiencies caused by disagreements among sponsors, investigators, and regulators. To address this issue, the Clinical Trials Transformation Initiative convened content experts and stakeholders to develop recommendations for pregnancy testing in clinical research based on currently available evidence. Recommendations included: 1) the study protocol should clearly state the rationale for pregnancy testing and the plan for handling positive and indeterminate tests; 2) protocols should include an assessment of the pregnancy testing plan advantages (reduced risk of embryo/fetal exposure) versus the burdens (participant burden, study team workload, costs); 3) protocols should assess the participant burdens regarding the likelihood of false negative and false positive results; 4) participant administered home pregnancy testing should be avoided in clinical trials; and 5) the consent process should describe the extent of knowledge about the study intervention’s potential risk to the embryo/fetus and the limitations and consequences of pregnancy testing. CTTI has also developed an online tool to help implement these recommendations.

## Introduction

Minimizing the risk of an unintended embryo/fetal exposure to a drug that could potentially cause birth defects is a major concern of the research and regulatory communities. For drugs studied under an Investigational New Drug (IND) application, non-clinical studies provide some information on safety but data on the effects on human development *in utero* are typically limited. Historically, the response within the research and regulatory communities was to exclude females of reproductive potential (FRP) from clinical trials. However, recognition of the ethical and scientific value of including FRP led to changes in policy [1], and FRP now participate in all phases of clinical research. Risk minimization is attempted by 1) mandating the use of effective contraception for defined periods relative to exposure and 2) testing for pregnancy prior to study entry and, in some cases, periodically throughout the study.

There are no evidence-based standards on appropriate methods for identifying early pregnancy among research study participants to minimize the risk of unintended embryo/fetal exposure. The dilemma of efficient and accurate diagnosis of early pregnancy challenges expert clinicians as well as dedicated researchers [2]. Different pregnancy tests offer varying sensitivity and specificity in detecting an early pregnancy as well as markedly different levels of convenience for participants (e.g., at-home versus going to a lab; blood draw versus urine sample). Multiple manufacturers offer multiple tests with different performance parameters (e.g., varying ability to detect pregnancy before versus after a missed menses) and different reporting styles (e.g., a positive or negative result versus a hormone level), leading to vastly different practices across the research endeavor. The lack of consensus about the optimal criteria for the type and timing of pregnancy testing leads to inconsistency among testing protocols and inefficiencies in safety monitoring of research participants. It also raises potential conflicts between investigators, sponsors, and institutional review boards (IRBs) on minimizing the risk of exposure to subjects while also attempting to minimize burdens to study participants.

The Clinical Trials Transformation Initiative (CTTI; http://ctti-clinicaltrials.org), a public-private partnership established by Duke University and the U.S. Food and Drug Administration (FDA) in 2007 to identify and drive adoption of practices that increase the quality and efficiency of clinical trials, sought to develop evidence-based recommendations for pregnancy testing in clinical research that will enhance protection of research participants, reduce the risk of unintended embryo/fetal exposure, and incorporate the interests of all stakeholders—participants, researchers, sponsors, and regulatory bodies. The focus of this project is on preventing unintended embryo fetal exposures. For this reason, we discuss pregnancy testing in the context of preventing enrollment of pregnant women in clinical research [3]. Although we recognize the ethical imperative to conduct research in pregnant women expressly for better understanding how to safely care for them [4–5], that is not our objective here. Developing specific pregnancy testing recommendations that would fit all research situations is an unattainable task. Thus, we sought to identify fundamental principles that could be incorporated into the study design process to assist in the development of evidence-based protocols around pregnancy testing.

Within the context of avoiding unintended exposures after FRP are enrolled in a clinical trial, effective contraception is a key element. A thorough review of contraceptive options for FRP in clinical trials and the inherent clinical and ethical ramifications lies outside the scope of this analysis, although current practices were recently reviewed and highlight similar challenges in assessing and minimizing risk [6].

The goal of this paper is to summarize our project findings, specifically by 1) providing a review of the relevant background information necessary to assess the potential pregnancy-related risks of a trial; 2) introduce an evidence-based simulation model for evaluating these risks and the benefits of different testing protocols; and 3) propose recommendations for how to address this issue during protocol development based on current evidence and expert opinion.

## Materials and methods

CTTI projects utilize multistakeholder project teams that follow an evidence-based methodology to identify impediments to research, gather evidence to identify gaps and barriers, explore results by analyzing and interpreting findings, and finalize solutions by developing recommendations and tools [7]. The CTTI Pregnancy Testing in Clinical Trials Project Team comprised representatives from academia, the pharmaceutical industry, the FDA, and patient representatives and advocacy organizations (https://www.ctti-clinicaltrials.org/projects/pregnancy-testing). The study was reviewed by the Duke University Health System Institutional Review Board (IRB) and was granted a declaration of exemption from IRB review (Pro00045452). Written informed consent was not required. Potential participants received an explanation of the electronic survey and could choose to decline or complete the survey. The study protocol can be accessed at http://dx.doi.org/10.17504/protocols.io.rjxd4pn.

### Survey

The project team developed and conducted an internet-based survey to assess which factors are currently considered when designing pregnancy testing plans for clinical trials. The intent of the survey was to better understand current risk assessment and to frame the discussions at the expert meeting rather than to draw general inferences regarding pregnancy testing within the research community at large.

The project team developed the survey content collaboratively by creating and revising content on monthly teleconferences. The survey (S1 Appendix) included a maximum of 48 questions, including 35–45 multiple choice questions about five different clinical trial scenarios, and an open-ended question about other factors considered in developing pregnancy testing plans. Questions about clinical trials scenarios asked respondents to provide an estimate of the acceptable risk of a false negative pregnancy test resulting in an embryonic exposure to study drug; rank the importance of negative predictive value, subject burden, study team burden, and testing cost; and describe the basic type of testing protocol recommended for that scenario. The project team group identified key stakeholders involved in the design, conduct, and evaluation of clinical trials to participate in the survey. Potential participants were identified by requesting referrals from the project team and from CTTI Steering Committee (SC) representatives (https://www.ctti-clinicaltrials.org/about-us_main/organization/steering-committee) from academic medical centers, pharmaceutical industry, clinical research organizations and sites, institutional review boards, and government organizations. Prior to distribution, two clinical trial investigators reviewed, tested, and provided suggestions for modifications to the survey. The content was entered into online survey software (Qualtrics 2009; Provo, Utah, USA) and was emailed to the selected audience.

### Model

In parallel with conducting the survey, the academic team leader created a probabilistic computer simulation model to estimate expected rates of false negative and false positive pregnancy tests during clinical trials as a function of population-specific parameters including subjects’ age and contraceptive use, type of pregnancy test, frequency of testing, and timing of testing relative to menses. Additional model parameters include hysterectomy status, menopausal status, menstrual cycle characteristics, pregnancy outcome probabilities, contraceptive effectiveness (typical use), hCG levels in non-pregnant women, hCG levels in pregnancy, sensitivity of hCG assays and probability of detecting symptoms in the absence of testing (S2 Appendix). The model was created using modeling software (TreeAge Software Inc., 2012; Williamstown, MA, USA) with probabilities based on data from the published literature, regulatory filings, and package inserts (S2 Appendix References). A simplified deterministic version of the model was subsequently constructed in Microsoft Excel to facilitate interactive use via internet. The overall structure and output of the simplified model is similar to the more complicated model, except that the simplified model does not provide confidence intervals for estimates, while allowing much faster calculations in the on-line setting. Since the goal of the online tool is to facilitate decisions about pregnancy testing protocols for a trial by providing reasonable estimates of the effect of different pregnancy testing choices on number of pregnancy test outcomes in a trial, the trade-off of improved end-user experience over estimating quantitative uncertainty was considered reasonable.

### Expert meeting and recommendations development

Subsequently, CTTI convened a 2-day expert meeting (July 15–16, 2013, Bethesda, MD, USA) to present survey findings and computer simulation model results, discuss practices and challenges in assessing the acceptable risk of pregnancy and implementing a pregnancy testing protocol for a clinical trial, solicit additional feedback, and draft consensus recommendations. The meeting utilized a combination of moderated full group and breakout group sessions utilizing prepared questions and open discussion (https://www.ctti-clinicaltrials.org/files/pregnancytesting-meeting_agenda.pdf). The project team then reconvened in monthly teleconferences to finalize project recommendations [7] based on the data gathered and discussions from the expert meeting.

## Results

### Survey

Summary results are provided in Table 1. Full survey results are available at http://dx.doi.org/10.17504/protocols.io.rj8d4rw. Survey questions included five different risk scenarios, ranging from a 12-month Phase 3 trial of a thalidomide derivative to a Phase 2 trial of an antiemetic with exposure limited to the perioperative period. Across this range of research scenarios with varying levels of risk to an early pregnancy, approximately half of respondents (range from 48–69%) chose the most conservative levels of maximal acceptable risk of pregnancy given (0.001% and 0.01% combined). Respondents consistently rated the negative predictive value (meaning a negative test rules out a pregnancy with a high degree of certainty) of a test as the most important consideration, followed by minimizing the patient burden of a testing protocol. Respondents expressed variability in acceptable testing method based on the degree of known embryo/fetal risk of study drugs, with an average 54.6% (37–86%) selecting serum testing across the scenarios. The overwhelming majority, an average of 93.4% (90–97%), did not consider home testing acceptable, independent of the level of known embryo/fetal risk.

**Table 1:** Survey summary findings.

Response rate	67% (39 participants of 58 invited)
Organization type of respondents (more than one response could be selected)	16 (44%) academic medical center
	13 (33%) industry
	5 (13%) institutional review board
	3 (8%) clinical research organization
	3 (8%) government
	2 (5%) non-academic research site
Most important testing protocol characteristics for all clinical scenarios	High negative predictive value (average importance rating range 4.0–4.7)
(Scale of 1 = Not Important at All to 5 = Extremely Important)	Low patient burden (average importance rating range 3.0–3.2)
Percent of respondents amenable to participant-administered home pregnancy testing (independent of level of embryo/fetal risk)	3%–10%

### Model

The model was created to estimate the outcomes of different pregnancy testing protocols. As anticipated, when a theoretical sample population is used, the risk of pregnancy occurring during a trial decreases with an increasing average age of subject population. As a study population ages, there are fewer females of reproductive potential due to an increased prevalence of infertility-associated diseases (such as pelvic inflammatory disease), hysterectomy, decreased ovarian reserve, or menopause. In addition, there is greater use of highly effective contraceptive methods, particularly sterilization. Timing of testing was noted to be crucial, with false negatives being higher when testing is not performed relative to the menstrual cycle. Of note, overall estimated absolute differences in false negative tests comparing serum and urine tests are quite small. In younger women, who have a higher probability of pregnancy, the absolute difference is 5/10,000. In women closer to menopause, the difference is 3/10,000. This suggests that using a serum test detects two pregnancies per 10,000 women that would otherwise be missed if a urine test were used. On the contrary, false positive results increase with age due to increases in pituitary hCG during and after menopause [8–9], but only when the threshold for a positive serum test is at an hCG concentration of 5–19 IU/L (above the standard cutoff of 5 IU/L); given the sensitivity of most commonly used urine tests, false positive results are essentially only an issue with serum testing. These general model findings reflect the value of the information that can be gained and applied to a decision-making process regarding testing algorithms within a clinical trial.

### Key points from expert meeting

A 2-day expert meeting was convened in July 2013, with 41 participants attending, including representatives from industry, academia, patient advocacy organizations, and government agencies. A list of meeting participants, the agenda, a meeting summary, and presentations can be accessed at www.ctti-clinicaltrials.org/what-we-do/investigational-plan/pregnancy-testing/meetings. Formal presentations guided the conversation and are summarized in the following pages.

#### Universal “minimal acceptable risk” is not definable

“Minimal acceptable risk” is the acceptable probability of having an unintended embryo/fetal exposure within a clinical trial. The estimation of this threshold is complex and involves multiple variables, both objective (the age of the study population, their underlying medical conditions and baseline fertility) and subjective (harm/cost of an embryo/fetal exposure). The objective component should be arrived at in a rational manner based on some of the background and principles outlined below. Although study designers take this risk into account as they design the pregnancy testing plan, and inform potential participants about known or unknown risks to the best of their ability during the consent process (see Recommendations), it is participants who ultimately accept this risk by agreeing to participate in the study [5].

#### Teratogens and teratogenic risk

Teratogens are defined as any substance or process that interferes with normal prenatal development. Although teratogens can be present in the form of maternal disease (e.g., diabetes) or infection (e.g., rubella), within the context of clinical trials, we are referring to exogenous teratogens, mainly medications. Early exposure to teratogens can interfere with normal embryologic development such that a miscarriage occurs. Within the clinical trial setting this is of concern given the high and often unappreciated background risk of early pregnancy loss in the general reproductive-age population, estimated at 15%–20% [10–12]. Determining the actual cause of a miscarriage is often complex or impossible.

Although preclinical testing of a new medication may identify some safety risks, many phase 2 and phase 3 trials start with a high degree of uncertainty about the specific risks of teratogenicity in humans. These risks can be minimized by preventing or minimizing exposure to possible teratogens through 1) pregnancy testing prior to enrollment to exclude pregnant women from a trial; 2) pregnancy testing prior to study intervention; 3) consistent use of an effective contraceptive method to prevent pregnancy during a trial; 4) stopping study drug once pregnancy is detected; and/or 5) providing appropriate counseling.

#### Measurement of human chorionic gonadotropin (hCG)

The hCG molecule, a member of the glycoprotein hormone family, has alpha (α) and beta (β) subunits that are similar to other proteins in that family (i.e., luteinizing hormone [LH], follicle stimulating hormone [FSH], and thyroid stimulating hormone [TSH]). The beta subunit is more discrete than the alpha subunit, but still shares many similarities and even receptors with LH. Concentrations of hCG progressively increase in early pregnancy, usually peaking by around 10 weeks. Normal variants of β-containing hCG found in both serum and urine include intact hCG, nicked hCG, free hCG, and hyperglycosylated hCG. Hyperglycosylated hCG is elevated in early pregnancy. Another β-associated variant, β-core fragment, is found only in urine. A recognition of hCG variants and their different times and locations of expression is crucial to understanding hCG immunoassays, described in Table 2:

**Table 2:** Pregnancy test characteristics.

Test Type	Location	Typical Sensitivity (IU/L)*
Qualitative		
• Urine	Home/Point-of-care	20–25
• Serum	Lab	~10
Quantitative		
• Serum	Lab	2 (cutoff 5 IU/L)

*Reviewing the specific package inserts of the assay used is strongly recommended because testing sensitivity may vary between tests in addition to the way sensitivity is described. These values are based on: Snyder 2005 [9], Cervinski MA, Lockwood CM, Ferguson AM, et al. Qualitative point-of-care and over-the-counter urine hCG devices differentially detect the hCG variants of early pregnancy. Clin Chim Acta. 2009; 406(1–2):81–5. 10.1016/j.cca.2009.05.018., Sturgeon CM, Berger P, Bidart JM, et al. IFCC Working Group on hCG. Differences in recognition of the 1st WHO international reference reagents for hCG-related isoforms by diagnostic immunoassays for human chorionic gonadotropin. Clin Chem. 2009;55(8):1484–91. 10.1373/clinchem.2009.124578., and Furtado LV, Lehman CM, Thompson C, Grenache DG. Should the qualitative serum pregnancy test be considered obsolete? Am J Clin Pathol. 2012; 137(2):194–202. 10.1309/AJCPH1PJSA9TWYOZ.

Currently, there is a lack of standardization across assays. This is partly because although tests traditionally detect intact hCG, other forms of hCG may also be detected by many of the assays, and there are inconsistencies in how the variants are reported in test results [13–16]. This variability makes comparing specificity across tests complicated, which is of great concern in clinical trials, given the desire to accurately rule out pregnancy with a negative test. Another important issue to consider regarding detection of variants is that the possibility of false negative results arises when high levels of βhCG core are present [17]. Such high levels are typically seen at around 8 weeks of pregnancy and not at the early stages of greatest concern to clinical researchers. However, investigators should be aware of this possibility, because it could lead to enrolling a woman with a relatively advanced pregnancy. The need for clinical correlation to interpret the test result in light of menstrual data and physical findings, and consistent evidence of observer variability in interpretation of consumer pregnancy test results [18–19], suggest that this is best done by investigators and not by study participants.

Differences in analytical sensitivity may lead to significant variability across tests, including the range at which a given test result is “indeterminate.” Thus, the probability of a positive or indeterminate result may vary between tests. In the context of clinical trials, increased numbers of indeterminate results could cause unnecessary delays in enrollment, delays in administration of study interventions, or participant withdrawal. In addition, in any context (e.g., literature; product labeling) it is important to note the definition used for hCG sensitivity. For example, in some contexts, “cutoff” or “sensitivity” might reflect the level at which 50% of samples are positive; for others, it may reflect the level at which all samples are positive. However, even if current hCG cutoffs are maintained, recognition of variability between brands and assays is important to consider, especially in multi-site trials where different tests may be used across sites, resulting in differing abilities to detect pregnancy.

Clinical sensitivity refers to the accuracy of the test in relation to the timing of testing. When a pregnancy can be detected is highly correlated to the menstrual cycle. Even the most sensitive test cannot detect an early pregnancy before hCG production is initiated by the trophoblast. Thus, there is minimally an 8–10 day window between conception and implantation when any pregnancy test will be negative. Most home pregnancy tests can detect more than 50% of pregnancies by the expected day of menses [19]. In women undergoing blastocyst transfer, a serum test can detect pregnancy 3 days before expected menses, but whether this is generalizable to spontaneous conceptions and all serum tests is unclear [20]. Although the initiation of hCG production is consistent across patients, the rate of rise, much like menstrual cycles, can vary significantly [21]. Due to variation in cycle length, testing 15 days after the LH surge (detected by urine or serum evaluation) results in less variability in pregnancy detection than timing a test at the expected time of menses. However, in the study setting, this is probably not feasible. Clinical trial pregnancy testing plans are often designed such that testing is timed to a participant’s menstrual cycle. However, given the variability in cycle length across women, estimating the appropriate time to test is not straightforward.

The value of clinical specificity arises in the setting of interpreting the presence of persistently low hCG levels, resulting in false positive results. These results are categorized as “classic” and “biological,” both of which have a profound impact on trial continuation. “Classic” false positives typically arise in the setting of interfering antibodies, pituitary hCG, and exogenous hCG. Interfering antibodies usually only occur with serum tests. Often they can be identified (and a true negative result obtained) by running dilution studies, using blocking antibodies, repeating the test with a different assay, or testing a urine sample. Pituitary hCG, most commonly elevated in peri- and post-menopausal women, can interact with hCG assays and cause a false positive test [8–9]. Follow-up testing with FSH to confirm lack of ovarian function typically resolves the clinical conundrum [22].

Exogenous hCG (often from supplements) can also cause a positive test in the absence of pregnancy. However, the more common false positives are “biological” false positives. These come from conceptions that result in implantation, leading to hCG production, but are followed by spontaneous miscarriage by the time of the expected menses. These very early pregnancy losses (often called “chemical pregnancies”) are thought to affect 15%–20% of conceptions in the general reproductive-age population [10–12]. Any of these falsely positive results can compromise participant continuation in a clinical trial.

#### FDA regulation of pregnancy tests

Pregnancy tests are regulated by the FDA as Class II (moderate risk) medical devices and are “cleared” via the 510(k) process prior to marketing [23]. In this process, an applicant submits a premarket 510(k) submission to the FDA in which the applicant demonstrates “substantial equivalence” of the new device to a predicate (i.e., previously cleared) pregnancy test of their choice. Information to support 510(k) clearance for hCG tests typically includes method comparisons to a predicate device using clinical samples and demonstration of other performance characteristics such as detection limits/sensitivity, precision, recovery, linearity (for quantitative tests), testing for interference, and traceability and stability information. Examples of performance characteristics reviewed in this process can be found in FDA 510(k) Decision Summaries that can be accessed using the following link: http://www.accessdata.fda.gov/scripts/cdrh/cfdocs/cfPMN/pmn.cfm

Given the considerations discussed here, it is important for those selecting pregnancy tests for use in clinical research to be familiar with the specific performance demonstrated (i.e., review package inserts and/or medical literature) for the pregnancy tests being considered and consider whether only one brand or type of test should be used across sites. Further, as new information arises, evaluations to assess these effects may be performed by manufacturers of new pregnancy tests and/or reported in the literature.

## Discussion

We undertook this project to address a critical problem in the research community—lack of consistency and guidance in developing and implementing pregnancy testing safety plans. It became clear during our review and discussions that although there is an implicit goal to completely eliminate the risk of an unintentional exposure of an embryo or fetus to interventions occurring as part of clinical research, this goal is (a) not feasible, and (b) inconsistent with the wide range of current practice and general lack of formal consideration of both the risk of a given study and the likely outcomes of specific pregnancy testing protocols in specific patient populations. Developing an appropriate testing protocol not only requires understanding the specifics of test sensitivity and specificity, but also the timing of the test and the interpretation of results. There is a lack of formal data on pregnancies occurring during clinical trials. The pregnancy rate in the Phase 3 trials of the quadrivalent HPV vaccine was 17%, which, given the age range, inclusion criteria requiring ongoing sexual activity, and effectiveness rates of allowed contraceptive methods, likely represents the upper range [24]. In trials where women of reproductive age are likely to be in their 40’s, and where the disease process and treatment may affect fertility either directly (through effects on the reproductive system) or indirectly (through reducing the frequency of intercourse), pregnancy rates are likely to be much lower.

### Recommendations

Given the complexity and importance of the process of interpreting pregnancy test results, investigators, not study participants, are best suited to performing this task in clinical trials [8–22]. Pregnancy testing should therefore be restricted to a lab or clinic (as opposed to home) setting. We have also come to understand the cognitive dissonance that affects researchers and regulators regarding this issue. We strive for unattainably low levels of pregnancy risk, yet design pregnancy testing plans without elucidating or quantifying the risks inherent to the study or the testing plan. We use tests that vary in their sensitivity and specificity even within the same multisite trial.

Below, we propose several principles and tools to improve this process. Based on basic biological information, results from our stakeholder survey, preliminary model findings, and consensus at our expert meeting, we suggest general principles (Table 3) to guide the process of developing pregnancy testing plans in clinical trials. We also propose more specific recommendations (Table 4) and identify areas where further research could clarify uncertainties. These recommendations and an online calculator are intended to provide tools for stakeholders involved in developing or assessing pregnancy testing plans in clinical trials.

**Table 3:** General principles.

Define “minimal acceptable risk”	The “minimal acceptable risk” of an unintended exposure of an embryo or fetus occurring in a clinical research participant: Varies with each study; and Should be defined *a priori* by the investigators, in consultation with patients and other stakeholders
Basic epidemiologic and reproductive science, as well as current evidence, should guide pregnancy testing plan development, including:	Characteristics of the target patient population, such as: Age distribution, which will affect background risk of pregnancy, miscarriage, congenital anomalies, and pregnancy complications, and contraceptive methods; and Effects of the underlying disease and/or study treatments on fertility, pregnancy complications, contraindications to specific contraceptive methods, etc. Basic reproductive biology: Timing of ovulation, conception, implantation, menses; Mechanisms of action of different contraceptive methods; and Incidence of chemical pregnancies/early pregnancy loss Basic hCG endocrinology: Patterns of hCG in early pregnancy; Implications of different variants in pregnancy testing; and Causes of false positive results Performance of available hCG tests: Comparison of claimed sensitivity & specificity for detection of hCG; and Consider using only one brand or type of test across all sites in a multicenter trial Estimation of the likelihood of false positive and false negative pregnancy tests of different testing plans (type and timing of tests) given the above considerations

**Table 4:** Specific recommendations for pregnancy testing in clinical trials.

**Recommendations for investigators and sponsors in developing a clinical trial protocol**:	
The protocol should clearly state the specific purposes of pregnancy testing in the research study	Prevent or minimize embryo/fetal exposures to study drug/intervention by: Confirming non-pregnant state at time of enrollment and, if applicable, prior to any subsequent exposures; and Detecting early pregnancies to determine whether to continue participation in the study
The protocol should describe the procedure for handling positive or indeterminate pregnancy tests	Define a positive test as it relates to the specific pregnancy testing plan (actual measured level of hCG that is considered positive) Define an indeterminate test (level of hCG, which will vary by study population age and underlying medical condition, and type of pregnancy test used) Define: Procedures for follow-up testing and evaluation of both positive and indeterminate tests and procedures for continuing, holding, or stopping study interventions and appropriate medical follow-up in the event of positive or indeterminate test results (based in part on the potential embryo/fetal risks of exposure to study interventions and the potential benefit to the participant from continued study participation)
**Recommendations for investigators when developing a pregnancy testing plan**	
Assess the balance of the pregnancy testing plan advantages (reduced risk of embryo/fetal exposure) versus burdens (participant burden, study team workload, costs).	This can be done using formal quantitative or qualitative methods. Formal quantitative methods incorporating parameters including age of study population, type of contraceptive methods used by the study population, type of pregnancy test used and its detectable threshold of hCG, and the proposed timing of testing during the menstrual cycle) to estimate: The negative and positive predictive values of a proposed testing strategy; and The absolute differences in exposures prevented based on variable testing options Alternatively, a semi-quantitative or qualitative assessment of risks and burdens considering the same factors.
Assess participant burdens regarding the likelihood of false negative results and unintentional embryo/fetal exposure, and likelihood of false positive results	Invasiveness of testing (serum versus urine tests) Timing of testing (random versus timed to the menstrual cycle) and study interventions Implications of false positives (repeat testing, delay in receipt of study interventions, study withdrawal, anxiety/worry) for the patient
Avoid participant-administered home pregnancy tests in clinical trials	Although patient-administered tests offer convenience to both participants and study staff, disadvantages include Consistent evidence of observer variability in interpretation of consumer pregnancy test results Potential for emotional distress in event of participant-read false negative result and subsequent embryo/fetal exposure Potential for desire to continue in study affecting interpretation of ambiguous test results
Recommendations for participant education during the consent process*	
Clearly articulate extent of knowledge about potential embryonic or fetal risks from exposure to study intervention	In addition, acknowledge in the informed consent process that: Pre-clinical testing on animals may not fully inform assessment of risk in humans; and Even when clinical trial and/or post-market data are available, overall knowledge about potential embryo/fetal risks may be minimal
Clearly explain the limitations and consequences of pregnancy testing to participants during the consent process	Potential for false negatives—No available test will detect 100% of pregnancies Potential for false positives—The possibility of a positive test in non-pregnant participants—this varies based on patient age, other conditions, and type of test The implications of a positive or indeterminate test for study participation: What additional tests/procedures will be performed to confirm a pregnancy? Who decides on whether to continue or terminate study participation? What criteria will be used to make that decision? How will pregnancy outcomes be followed? Who is responsible for ensuring patients will have appropriate medical follow up?

* Acknowledging efforts to simplify the informed consent form, these recommendations apply to the consent process. For example, a separate concise information sheet could be created for females of reproductive potential (FRP) or if desired included in the consent form as a separate page for FRP only.

### Online tool

Based on the model developed as part of the CTTI project, CTTI created an interactive, evidence-based simulation model for estimating the outcomes (e.g., false negatives, false positives) of different testing protocols. The tool is available as an online calculator (https://connects.ctti-clinicaltrials.org/preg_test/index) for estimating the probability of unintended embryonic or fetal exposures with different testing strategies. Users specify the expected number of women in the trial, an estimate of the age distribution, and the duration of the study. Based on the age distribution, the model estimates the number of women of childbearing potential, and the probability of pregnancy during the course of the trial under scenarios of (a) no contraception, (b) population-based distributions of contraceptive methods, and (c) a requirement of highly effective contraceptive methods only. Under an assumption that pregnancy testing is performed randomly in reference to subjects’ menstrual cycles, the model then estimates the number of true negative and positive pregnancy tests, false negative pregnancy tests, and, in the case of serum testing, false positive results at initial screening and during subsequent follow-up. By comparing the number and type of expected results using tests with different thresholds, investigators, regulators, and sponsors can make informed decisions about the trade-offs involved with using a specific pregnancy test in a given study population (note that the model does not estimate individual pregnancy risk). The model is evidence-based, with documentation of underlying sources for the estimates and updated on a regular basis as evidence changes.

### Evidence gaps

Although data are currently available to inform a more rational approach to developing of pregnancy testing protocols in clinical trials, there are evidence gaps to be addressed that could lead to more efficient and possibly less risky protocols. These gaps include a lack of understanding about currently used pregnancy testing plans, setting appropriate test standards, and incorporating patient preferences.

We lack a clear understanding of what happens across trials when there are false negatives (and therefore unintended exposures) or false positives (and unnecessary withholding of medication or withdrawal from a study). We do not know the magnitude or severity of the problem. However, studies of drugs evaluated as part of an IND application may contain some of this data. By leveraging this information and identifying associations between population characteristics, type of test, timing of test, and outcome (false positive or false negative), we may be able to draw limited conclusions about testing strategies by population. Similarly, data gathered from post-approval sources such as existing pregnancy registries and adverse events databases might provide much-needed information about levels of risk within certain drug classes or exposures to assist in more accurately tailoring testing protocols.

Current regulatory guidelines allow for variation in analytic sensitivity between tests, not only in cutoff values but in hCG subunits detected. It is unclear what the appropriate cutoffs should be, and how currently marketed versus newly approved tests should be compared. Further investigation into the effects of changes in the approval/clearing process and comparability between tests is necessary to allow for more accurate determination of appropriate test selection.

Patient preferences are a crucial yet poorly understood and often ignored aspect of pregnancy testing in clinical trials. The informed consent process should include the provision of information regarding potential risks of an unintended exposure, as well as the possible implications of a positive test. Although a research protocol may require withdrawal from the study, if pregnancy is detected, participants—especially those receiving much-needed medications—may not want to withdraw, even at the risk of harm to a new pregnancy. Determining what autonomy participants should have in the setting of a positive pregnancy test is not completely clear. The ethical aspects of this scenario need further exploration to allow for designing protocols that properly balance investigator liability and participant autonomy.

## Conclusion

Based on evidence review and key stakeholder input across academia, industry, and regulatory agencies, we propose evidence-based recommendations for developing pregnancy testing protocols in clinical trials. These guidelines aim to balance the potential risks of embryonic or fetal exposure with unnecessary burdens on participants and inefficiencies within the research process. Coupled with an interactive model (to be released simultaneously with the recommendations) that can be tailored to the specific population, these recommendations will allow for a more rational and transparent approach to the potential harms and benefits of minimizing possible embryo/fetal risks while maximizing benefits of clinical trials.

## Supporting information

S1 Appendix CTTI pregnancy testing in clinical trials project survey. (PDF)

S2 Appendix CTTI pregnancy testing model description. (PDF)
