# Supplementary material for: Evidence-based pregnancy testing in clinical trials: Recommendations from a multi-stakeholder development process
Source: PLoS One. 2018 Sep 12;13(9):e0202474. doi: 10.1371/journal.pone.0202474 (PMC6135366; doi:10.1371/journal.pone.0202474)
Supplement: S1 Appendix — (PDF) [file pone.0202474.s001.pdf]

S1 Appendix. CTTI pregnancy testing in clinical trials project survey.

First, we would like to ask a couple of background questions about you.

Which of the following best describes your organization? Please check all that apply.

- ❑ Academic Medical Center
- ❑ Non-academic Clinical Research Site
- ❑ Clinical Research Organization
- ❑ Institutional Review Board
- ❑ Industry
- ❑ Government (FDA, NIH, VA)
- ❑ Other, please specify ____________________

If applicable, which do you consider your PRIMARY area of clinical experience/expertise?

- ❍ Women's health/obstetrics & gynecology
- ❍ Family medicine
- ❍ Internal medicine (including subspecialties)
- ❍ Surgery (including subspecialties)
- ❍ Neurology/psychiatry
- ❍ Nursing
- ❍ Pharmacology or pharmacy
- ❍ Other, please specify ____________________

Before continuing on to the main part of the survey, we would like to provide you some background.

The main purpose of pregnancy testing as part of a clinical research protocol is to minimize risk to an embryo or fetus from exposure to potentially harmful study interventions. The specific goal of testing may be to prevent exposure completely (by testing prior to any study interventions), or to minimize the duration of exposure during early pregnancy (by periodic testing during the study).

The negative predictive value (NPV) of a particular pregnancy testing protocol is the probability that a negative test result in a given patient population represents a true negative pregnancy test. The higher the negative predictive value of the pregnancy testing protocol, the less likely a false negative pregnancy test result will occur and in turn, the less likely a prolonged unintended exposure of the study intervention to the embryo or fetus will occur.

The NPV of any pregnancy testing protocol is a function of the subject's baseline probability of pregnancy (primarily related to age) and contraceptive method. (For purposes of this survey, the term "highly effective contraception" refers to methods that, when used consistently and correctly, have a failure rate <1 per 100 woman-years. Examples include male or female sterilization, long-acting reversible contraceptives such as hormonal implants or intrauterine devices, or the combination of oral contraceptives and barrier methods). Negative predictive value is also affected by the following factors:

- The sensitivity of the assay used to detect human chorionic gonadotropin (hCG)
    - Serum tests are able to detect lower levels of hCG than urine tests
- The timing of the assay relative to when a pregnancy started
    - Neither serum nor urine tests will be positive if performed prior to implantation in the cycle in which a subject becomes pregnant

Different choices of tests (urine or serum assays), timing of tests relative to expected menses, and frequency of testing may result in different negative predictive values in different subject populations. Conversely, in a given population, there may be several potential testing protocols that result in similar negative predictive values, but have different implications in terms of potential burden on the subject, burden on the study team, and testing costs.

For the following questions, we will describe a clinical study scenario and ask you a series of questions about the acceptable risk of an unintended fetal exposure to a drug, the importance of several factors going into clinical study design, and the type of pregnancy testing protocol that you would recommend.

Scenario: A Phase III study of a new analog of thalidomide (a drug known to be associated with a very high risk of birth defects). Study drug is administered for 12 months. Assume that all subjects are required to use two methods of highly effective contraception.

Would you recommend excluding pregnant women from this study?

- Yes
- No

If No Is Selected, Then Skip to End of Block

Please answer some additional questions about the previous scenario, restated below

A Phase III study of a new analog of thalidomide (a drug known to be associated with a very high risk of birth defects). Study drug is administered for 12 months. Assume that all subjects are required to use two methods of highly effective contraception.

In your opinion, what is the acceptable risk of a false negative pregnancy test resulting in exposure to study drug to the embryo or fetus?

- Less than 1 in 100,000 (0.001%)
- Less than 1 in 10,000 (0.01%)
- Less than 1 in 1,000 (0.1%)
- Less than 1 in 100 (1.0%)

In this scenario, how important is each of the following considerations?

	Not important at all	Not very important	Moderately important	Very important	Extremely important
Negative predictive value (minimizing false negatives)	❍	❍	❍	❍	❍
Burden on subjects	❍	❍	❍	❍	❍
Burden on study team	❍	❍	❍	❍	❍
Testing Costs	❍	❍	❍	❍	❍

For this scenario, which type of pregnancy test would you recommend using? (Choose 1)

- ❍ Serum hCG performed in lab
- ❍ Urine hCG performed in lab
- ❍ Point of care urine hCG performed by study personnel
- ❍ Home test performed by patient

For this scenario, how do you recommend scheduling the pregnancy test(s)? (Choose 1)

- ❍ Whenever convenient for subject
- ❍ Timed to expected menses

Please answer a few final questions about the previous scenario, restated below

A Phase III study of a new analog of thalidomide (a drug known to be associated with a very high risk of birth defects). Study drug is administered for 12 months. Assume that all subjects are required to use two methods of highly effective contraception.

For this scenario, at which study time points do you recommend a pregnancy test be performed? (Choose 1)

- ❍ Before study only
- ❍ Before and during study
- ❍ Before, during, and after study exposure completed

The final two questions on this page are only shown if applicable to the answer above.

Prior to study drug administration, when do you recommend testing for pregnancy? (Choose 1)

- ❍ At screening visit
- ❍ Immediately before administering study drug
- ❍ At screening visit and immediately before administering study drug

How often do you recommend testing for pregnancy during the study?

- ❍ Every month
- ❍ Every other month
- ❍ Every three months
- ❍ Other (Please Specify) ____________________

How long after study exposure is completed do you recommend continuing to perform pregnancy testing?

- ❍ 1 month
- ❍ 2 months
- ❍ 3 months
- ❍ 6 months
- ❍ 12 months

Scenario: A Phase III study of a new indication for a currently marketed drug administered for 6 months, currently classified as Pregnancy Category C (the relative risks and benefits in humans are unknown, because of positive data in animals only, no human data, or no data at all). Assume subjects are using a highly effective method of contraception as specified by the protocol.

Would you recommend excluding pregnant women from this study?

- Yes
- No

If No Is Selected, Then Skip to End of Block

Please answer some additional questions about the previous scenario, restated below

A Phase III study of a new indication for a currently marketed drug administered for 6 months, currently classified as Pregnancy Category C (the relative risks and benefits in humans are unknown, because of positive data in animals only, no human data, or no data at all). Assume subjects are using a highly effective method of contraception as specified by the protocol.

In your opinion, what is the acceptable risk of a false negative pregnancy test resulting in exposure to study drug to the embryo or fetus?

- Less than 1 in 100,000 (0.001%)
- Less than 1 in 10,000 (0.01%)
- Less than 1 in 1,000 (0.1%)
- Less than 1 in 100 (1.0%)

In this scenario, how important is each of the following considerations?

	Not important at all	Not very important	Moderately important	Very important	Extremely important
Negative predictive value (minimizing false negatives)	❍	❍	❍	❍	❍
Burden on subjects	❍	❍	❍	❍	❍
Burden on study team	❍	❍	❍	❍	❍
Testing Costs	❍	❍	❍	❍	❍

For this scenario, which type of pregnancy test would you recommend using? (Choose 1)

❍ Serum hCG performed in lab
❍ Urine hCG performed in lab
❍ Point of care urine hCG performed by study personnel
❍ Home test performed by patient

For this scenario, how do you recommend scheduling the pregnancy test(s)? (Choose 1)

❍ Whenever convenient for subject
❍ Timed to expected menses

Please answer a few final questions about the previous scenario, restated below

A Phase III study of a new indication for a currently marketed drug administered for 6 months, currently classified as Pregnancy Category C (the relative risks and benefits in humans are unknown, because of positive data in animals only, no human data, or no data at all). Assume subjects are using a highly effective method of contraception as specified by the protocol.

For this scenario, at which study time points do you recommend a pregnancy test be performed? (Choose 1)

- ❍ Before study only
- ❍ Before and during study
- ❍ Before, during, and after study exposure completed

The final two questions on this page are only shown if applicable to the answer above.

Prior to study drug administration, when do you recommend testing for pregnancy? (Choose 1)

- ❍ At screening visit
- ❍ Immediately before administering study drug
- ❍ At screening visit and immediately before administering study drug

How often do you recommend testing for pregnancy during the study?

- ❍ Every month
- ❍ Every other month
- ❍ Every three months
- ❍ Other (Please Specify) ____________________

How long after study exposure is completed do you recommend continuing to perform pregnancy testing?

- ❍ 1 month
- ❍ 2 months
- ❍ 3 months
- ❍ 6 months
- ❍ 12 months

Scenario: A 1 month Phase II study of a new intravenous antiemetic agent for prevention of postoperative nausea and vomiting, where exposure is limited to the perioperative period, drug elimination half-life is 6 hours, and preclinical studies show no signs of reproductive toxicity. Assume subjects are using a highly effective method of contraception as specified by the protocol.

Would you recommend excluding pregnant women from this study?

- Yes
- No

If No Is Selected, Then Skip to End of Block

Please answer some additional questions about the previous scenario, restated below

A 1 month Phase II study of a new intravenous antiemetic agent for prevention of postoperative nausea and vomiting, where exposure is limited to the perioperative period, drug elimination half-life is 6 hours, and preclinical studies show no signs of reproductive toxicity. Assume subjects are using a highly effective method of contraception as specified by the protocol.

In your opinion, what is the acceptable risk of a false negative pregnancy test resulting in exposure to study drug to the embryo or fetus?

- Less than 1 in 100,000 (0.001%)
- Less than 1 in 10,000 (0.01%)
- Less than 1 in 1,000 (0.1%)
- Less than 1 in 100 (1.0%)

In this scenario, how important is each of the following considerations?

	Not important at all	Not very important	Moderately important	Very important	Extremely important
Negative predictive value (minimizing false negatives)	❍	❍	❍	❍	❍
Burden on subjects	❍	❍	❍	❍	❍
Burden on study team	❍	❍	❍	❍	❍
Testing Costs	❍	❍	❍	❍	❍

For this scenario, which type of pregnancy test would you recommend using? (Choose 1)

- ❍ Serum hCG performed in lab
- ❍ Urine hCG performed in lab
- ❍ Point of care urine hCG performed by study personnel
- ❍ Home test performed by patient

For this scenario, how do you recommend scheduling the pregnancy test(s)? (Choose 1)

- ❍ Whenever convenient for subject
- ❍ Timed to expected menses

Please answer a few final questions about the previous scenario, restated below

A 1 month Phase II study of a new intravenous antiemetic agent for prevention of postoperative nausea and vomiting, where exposure is limited to the perioperative period, drug elimination half-life is 6 hours, and preclinical studies show no signs of reproductive toxicity. Assume subjects are using a highly effective method of contraception as specified by the protocol.

For this scenario, at which study time points do you recommend a pregnancy test be performed? (Choose 1)

- ❍ Before study only
- ❍ Before and during study
- ❍ Before, during, and after study exposure completed

The final two questions on this page are only shown if applicable to the answer above.

Prior to study drug administration, when do you recommend testing for pregnancy? (Choose 1)

- ❍ At screening visit
- ❍ Immediately before administering study drug
- ❍ At screening visit and immediately before administering study drug

How often do you recommend testing for pregnancy during the study?

- ❍ Every month
- ❍ Every other month
- ❍ Every three months
- ❍ Other (Please Specify) ____________________

How long after study exposure is completed do you recommend continuing to perform pregnancy testing?

- ❍ 1 month
- ❍ 2 months
- ❍ 3 months
- ❍ 6 months
- ❍ 12 months

Scenario: A Phase I study of a new chemotherapeutic agent in an advanced cancer patient population. The agent is administered once weekly for 4 weeks. Assume subjects are using a highly effective method of contraception as specified by the protocol.

Would you recommend excluding pregnant women from this study?

- Yes
- No

If No Is Selected, Then Skip to End of Block

Please answer some additional questions about the previous scenario, restated below

A Phase I study of a new chemotherapeutic agent in an advanced cancer patient population. The agent is administered once weekly for 4 weeks. Assume subjects are using a highly effective method of contraception as specified by the protocol.

In your opinion, what is the acceptable risk of a false negative pregnancy test resulting in exposure to study drug to the embryo or fetus?

- Less than 1 in 100,000 (0.001%)
- Less than 1 in 10,000 (0.01%)
- Less than 1 in 1,000 (0.1%)
- Less than 1 in 100 (1.0%)

In this scenario, how important is each of the following considerations?

	Not important at all	Not very important	Moderately important	Very important	Extremely important
Negative predictive value (minimizing false negatives)	❍	❍	❍	❍	❍
Burden on subjects	❍	❍	❍	❍	❍
Burden on study team	❍	❍	❍	❍	❍
Testing Costs	❍	❍	❍	❍	❍

For this scenario, which type of pregnancy test would you recommend using? (Choose 1)

- ❍ Serum hCG performed in lab
- ❍ Urine hCG performed in lab
- ❍ Point of care urine hCG performed by study personnel
- ❍ Home test performed by patient

For this scenario, how do you recommend scheduling the pregnancy test(s)? (Choose 1)

- ❍ Whenever convenient for subject
- ❍ Timed to expected menses

Please answer a few final questions about the previous scenario, restated below

A Phase I study of a new chemotherapeutic agent in an advanced cancer patient population. The agent is administered once weekly for 4 weeks. Assume subjects are using a highly effective method of contraception as specified by the protocol.

For this scenario, at which study time points do you recommend a pregnancy test be performed? (Choose 1)

- ❍ Before study only
- ❍ Before and during study
- ❍ Before, during, and after study exposure completed

Prior to study drug administration, when do you recommend testing for pregnancy? (Choose 1)

- ❍ At screening visit
- ❍ Immediately before administering study drug
- ❍ At screening visit and immediately before administering study drug

How often do you recommend testing for pregnancy during the study?

- ❍ Every month
- ❍ Every other month
- ❍ Every three months
- ❍ Other (Please Specify) ____________________

How long after study exposure is completed do you recommend continuing to perform pregnancy testing?

- ❍ 1 month
- ❍ 2 months
- ❍ 3 months
- ❍ 6 months
- ❍ 12 months

Scenario: A Phase II study of a new chemical entity, with no worrisome preclinical data on reproductive toxicity. Study drug is administered for 6 months. Assume subjects are using a highly effective method of contraception as specified by the protocol.

Would you recommend excluding pregnant women from this study?

- ❍ Yes
- ❍ No

If No Is Selected, Then Skip to End of Block

Please answer some additional questions about the previous scenario, restated below

A Phase II study of a new chemical entity, with no worrisome preclinical data on reproductive toxicity. Study drug is administered for 6 months. Assume subjects are using a highly effective method of contraception as specified by the protocol.

In your opinion, what is the acceptable risk of a false negative pregnancy test resulting in exposure to study drug to the embryo or fetus?

- ❍ Less than 1 in 100,000 (0.001%)
- ❍ Less than 1 in 10,000 (0.01%)
- ❍ Less than 1 in 1,000 (0.1%)
- ❍ Less than 1 in 100 (1.0%)

In this scenario, how important is each of the following considerations?

	Not important at all	Not very important	Moderately important	Very important	Extremely important
Negative predictive value (minimizing false negatives)	❍	❍	❍	❍	❍
Burden on subjects	❍	❍	❍	❍	❍
Burden on study team	❍	❍	❍	❍	❍
Testing Costs	❍	❍	❍	❍	❍

For this scenario, which type of pregnancy test would you recommend using? (Choose 1)

❍ Serum hCG performed in lab
❍ Urine hCG performed in lab
❍ Point of care urine hCG performed by study personnel
❍ Home test performed by patient

For this scenario, how do you recommend scheduling the pregnancy test(s)? (Choose 1)

❍ Whenever convenient for subject
❍ Timed to expected menses

Please answer a few final questions about the previous scenario, restated below

A Phase II study of a new chemical entity, with no worrisome preclinical data on reproductive toxicity. Study drug is administered for 6 months. Assume subjects are using a highly effective method of contraception as specified by the protocol.

For this scenario, at which study time points do you recommend a pregnancy test be performed? (Choose 1)

- ❍ Before study only
- ❍ Before and during study
- ❍ Before, during, and after study exposure completed

The final two questions on this page are only shown if applicable to the answer above.

Prior to study drug administration, when do you recommend testing for pregnancy? (Choose 1)

- ❍ At screening visit
- ❍ Immediately before administering study drug
- ❍ At screening visit and immediately before administering study drug

How often do you recommend testing for pregnancy during the study?

- ❍ Every month
- ❍ Every other month
- ❍ Every three months
- ❍ Other (Please Specify) ____________________

How long after study exposure is completed do you recommend continuing to perform pregnancy testing?

- ❍ 1 month
- ❍ 2 months
- ❍ 3 months
- ❍ 6 months
- ❍ 12 months

If there are other factors that contribute to your decision making about designing pregnancy testing protocols for clinical research that have not been included here, please describe them below. NOTE: Please do not include any identifying information in your response.

[Free text box provided]
