# Supplementary material for: Evidence-based pregnancy testing in clinical trials: Recommendations from a multi-stakeholder development process
Source: PLoS One. 2018 Sep 12;13(9):e0202474. doi: 10.1371/journal.pone.0202474 (PMC6135366; doi:10.1371/journal.pone.0202474)
Supplement: S2 Appendix — (PDF) [file pone.0202474.s002.pdf]

S2 Appendix. CTTI pregnancy testing model description.

Estimating Outcomes of Different Pregnancy Testing Protocols in Clinical Trials: Model Description

I. Purpose of the Model

Simulation models are often useful for comparing the estimated probability of relevant outcomes under different options, particularly when direct evidence is not available and where there are trade-offs (in terms of harms vs. benefits, costs, etc.) involved with the available options. Models can clarify the relative importance of uncertainty about specific components of the decision, as well as the importance of explicit or implicit assumptions made prior to making a decision.

The purpose of this model is to estimate the probability of relevant outcomes (false negative pregnancy tests, false positive pregnancy tests, and detected pregnancies) of different pregnancy testing protocols for either preventing exposure of an embryo/fetus to a study intervention (by identifying a previously unknown pregnancy) or minimizing the duration of exposure (by detecting a pregnancy as soon as possible so that study interventions can be stopped).

In the context of this expert meeting, the primary goal of the written and oral presentation of the model and results is to facilitate discussion of general principles to consider in making recommendations for pregnancy testing protocols. Secondary goals include:

- Obtaining feedback on the model inputs and structure; we discuss the rationale for the current version, but welcome specific suggestions on revisions or additional sources for data on relevant parameters
- Obtaining feedback on how a model-based analytic tool could be most useful for those designing and evaluating pregnancy testing protocols for clinical research (for example, an online resource which presented estimates of likely outcomes based on a range of preselected values for relevant variables such as subject age, contraceptive methods, and teratogenic risk, vs. an online resource which allowed a user to input specific values and obtain estimates, analogous to an online loan repayment calculator).

This document describes the data sources, assumptions, and derivations of the major inputs for the model. Results will be presented and discussed at the meeting.

II. Structure of the Model

We constructed a Markov model to simulate pregnancy testing of women of reproductive potential enrolled in a clinical trial excluding pregnant women (note that the model could also be applied to the evaluation of postmarketing pregnancy testing protocols) (Figure 1).

Figure 1: Schematic diagram of model. See text for description.

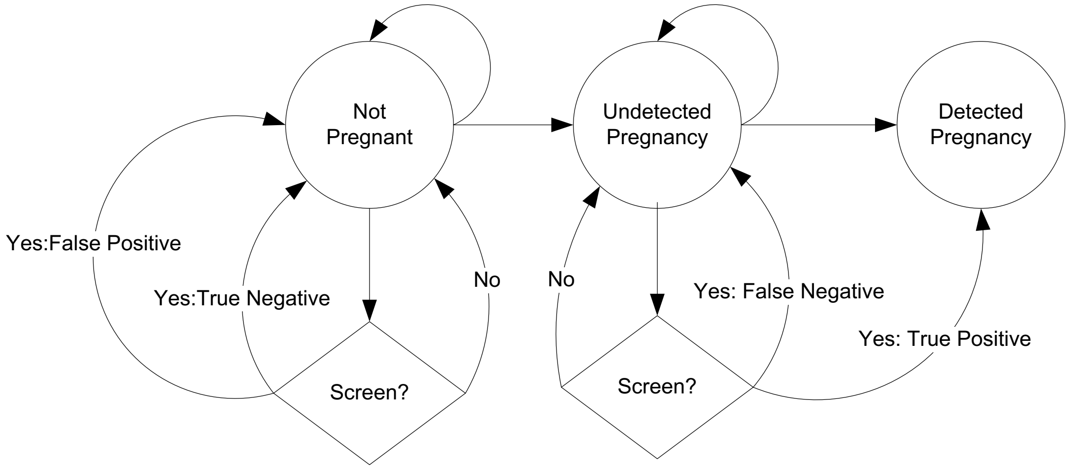

The model consists of 3 mutually exclusive states: Not Pregnant, Undetected Pregnancy, and Detected Pregnancy. In a Markov model, "subjects" can transition between states during a "cycle" or "stage." In this model, the length of one Markov cycle is 1 day. Each day, women in the Not Pregnant state can transition to the Undetected Pregnancy state, with a probability dependent on age, contraceptive method, and time in the menstrual cycle, or remain Not Pregnant. Women with an Undetected Pregnancy will ultimately have their pregnancy detected either through testing done for the clinical trial, or based on signs and symptoms of pregnancy.

Pregnancy testing is performed at the start of the simulation, with the probability of true negative, false negative, true positive, and false positive results dependent on the prior probability of pregnancy, the level of hCG in each state (a function of both subject age and the developmental stage of pregnancy), the assay sensitivity for detection of hCG, and the threshold used to define a "positive" test. Based on the duration of the "trial", follow-up testing can be performed at any interval.

The model is run as a stochastic microsimulation—multiple iterations of the model are run, with each iteration representing the experience of an "individual subject." Results from each "subject" are then summed and averaged across all iterations (typically, between 10,000 and 1,000,000). At the start of each iteration, a "subject's" age is drawn from one of the distributions described below. The value for age is then used to draw from distributions for age-specific prevalence of menopause and hysterectomy; "subjects" between the ages of 15 and 55 who are not post-hysterectomy or menopausal are then "assigned" a contraceptive method based on an age-specific distribution. In the base case analysis, we assume that the initial screen is performed independently of the menstrual cycle day; the potential impact of testing timed to the menstrual cycle is examined in subsequent analyses. After the

initial screen, the model is run for a minimum of 30 days and up to 365 days, with different screening intervals.

Specific types of statistical distributions were chosen based on the type of data and their fit to the reported data. Distributions included:

- *Normal*: the classic bell curve; mean and median values are equivalent. The potential range of a normal distribution is -∞ to ∞.
- *Gamma*: gamma distributions are frequently used as an alternative to the normal distribution when values below 0 are not possible.
- *Lognormal*: the natural logarithm of the values are normally distributed. Like gamma distributions, lognormal distributions have a minimum value of 0, and often used to describe values that have a particularly wide range (such as hCG levels). Lognormal distributions are also used with ratios, such as relative risks, odds ratios, and hazard ratios.
- *Beta*: beta distributions have a minimum value of 0 and a maximum of 1.0, and are used for values that are constrained by these limits (such as probabilities when there are only two possible outcomes, such as hysterectomy or no hysterectomy).
- *Dirichlet*: a Dirichlet distribution is a special form of the beta distribution used when there are more than two possible outcomes or states (such as contraceptive method).
- *Weibull*: Weibull distributions are survival functions which allow variation in the hazard function (i.e., the risk of an event can change with time, rather than remain constant).
- *Uniform*: The probability of any outcome or value is equal (for example, menstrual cycle day if testing is not timed relative to menses or other marker).

The model was built and all analyses performed in TreeAge Pro 2013 (TreeAge, Inc., Williamstown, MA). When necessary, conversion of published data to formats usable by the model was done in either Microsoft Excel (Redmond, WA: Microsoft Corp) or Parameter Solver v. 2.3 (Department of Biostatistics and Applied Mathematics, MD Anderson Cancer Center).

III. Model Parameters

All models require certain simplifying assumptions and decisions about which data sources to use to generate estimates for the model parameters. As much as possible, we erred on the side of overestimating pregnancy risk.

A. Subject Age

Table 1: Example conditions and age distribution

CONDITION	EXPOSURE	MEAN AGE (SD)	REFERENCE
Cystic acne	Isotretinoin	22.2 (8.2)	[1]
Epilepsy	Anti-epileptic drugs	36.7 (11.6)	[2]
Depression	SSRIs	44.0 (16.0)	[3]
Rheumatoid arthritis	Methotrexate	52.0 (13.1)	[4]

Age is the single most important determinant of pregnancy risk, and therefore the expected age distribution among trial subjects is the single most important predictor of the likelihood of pregnancy at the time of enrollment or during the trial.

Reported means and standard deviations for age were taken from 4 published trials of conditions that are relatively common in reproductive age women and frequently treated with drugs with known risks to

the fetus from exposure during pregnancy (Table 1), although not every study involved exposure to a potentially teratogenic agent. The studies were identified through a PubMed search using the terms "randomized trial" and the specific condition, and were chosen to provide examples of "typical" age ranges for trials in reproductive age women.

Because normal distributions with these values for mean and standard deviations often resulted in values outside of a plausible range (or the reported range of the study), we characterized the age of each subject population with lognormal distributions, which were a better fit than the alternative gamma distributions.

Although age is the most important determinant of risk of pregnancy among women of reproductive potential, the probability of NOT being at risk of pregnancy is also dependent on age, since both prevalence of hysterectomy and menopause vary with age. Thus, the age distribution in a given clinical research study will affect not only the probability of pregnancy *among women who are physiologically able to become pregnant*, but also the *overall* probability of pregnancy in the study.

B. Hysterectomy Status

Age-specific hysterectomy prevalence was estimated directly from the 2010 Behavioral Risk Factor Surveillance System (BRFSS), a population-based survey.[5] We used total population estimates. Because hysterectomy rates vary dramatically among racial/ethnic groups (in particular, African-American women are more likely to undergo hysterectomy at younger ages than other groups), race-specific estimates might be important for specific conditions where prevalence or incidence varies by race/ethnicity.

Table 2: Age-specific Hysterectomy Prevalence

	Age						
	**18-24**	**25-29**	**30-34**	**35-39**	**40-44**	**45-49**	**50-54**
Prevalence (95% CI)	0.4% (0.2-0.7)	1.8% (1.3-2.3)	4.0% (3.4-4.6)	7.1% (6.4-7.8)	12.6% (11.8-13.4)	19.4% (18.6-20.3)	27.9% (27.0-28.8)

C. Menopausal Status

Figure 2: Estimated age-specific prevalence of menopause

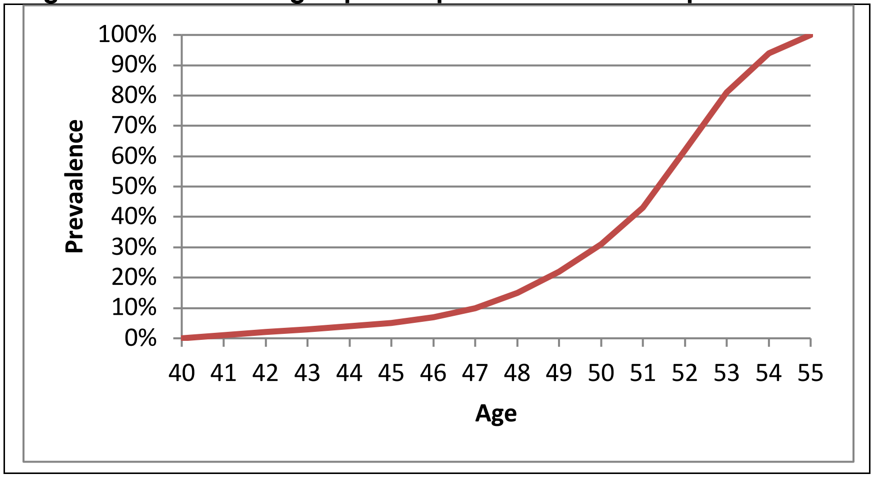

Figure 2 depicts the estimated age-specific prevalence of menopause in women with an intact uterus, derived from published data from the Study of Women's Health across the Nation (SWAN).[6] Estimates for ages 41-52 are based on the published Kaplan-Meier curve in Gold et al,[6] while estimates for ages 53-55 are based on fitting a Weibull distribution to the survival function based on the reported median age at menopause for the

entire population (51.4 years) and the estimated cumulative probability of menopause at age 52 based on the published K-M curve (52 years). We assumed that all women would be functionally menopausal at age 55, which was consistent with the estimated cumulative probability based on the Weibull distribution (99.2%). The published data from SWAN does not provide sufficient information to estimate confidence intervals around the age-specific prevalence. We also did not incorporate variation in age at menopause based on race/ethnicity, smoking history, or other factors, although, again, these could be readily incorporated if appropriate for a particular scenario.

D. Menstrual Cycle Characteristics

We used recent estimates of the duration and variability of the menstrual cycle among healthy premenopausal women[7](Table 3):

Table 3: Menstrual cycle characteristics

Parameter	Mean (95% CI)	Distribution
Cycle length (days)	27.7 (23-32)	Normal
Within-individual cycle-to-cycle variation (days)	2.8 (0.8-6.2)	Gamma
Length of luteal phase (days from serum LH peak)*	14.2 (10-18)	Normal
Within-individual cycle-to-cycle variation (days)	2.6 (0.3-9)	Lognormal

*Study reported time relative to urinary LH peak; we assume serum peak is one day earlier

At the start of each "individual" simulation, a cycle length is drawn from the cycle length distribution, and a value for cycle variability is also drawn. For simulations that last longer than one menstrual cycle, days are added or subtracted to the cycle length based on the variation (i.e., if the variability value is 2, the range of possible values is the initial cycle length value ± (2/0.5) days. Similar calculations are done for luteal phase length and variability. Within each cycle, the day of the serum LH surge was calculated as the cycle length minus the luteal phase length. This method allows for both between and within-individual variation in menstrual cycle length, while allowing appropriate modeling of day-specific hCG levels in the event of pregnancy.

Hormonal contraceptives affect both the probability of ovulation and menstrual patterns. For oral contraceptives, rings, and patches, we assigned a fixed cycle length of 28 days, and a fixed luteal phase length of 14 days. For simplicity, we did not attempt to model the effects of long-acting hormonal contraceptives on bleeding patterns; we did assume that protocols for continued testing in women using these methods would occur at fixed intervals in all cases, rather than being timed to menses.

We did not attempt to model age-specific variability in menstrual cycle length, although this is potentially an important consideration in women as they approach menopause.

E. Age-specific Conception Probability

Estimates of the cycle-specific probability of a detected pregnancy in couples not using contraception are available from a variety of sources, and include couples using natural family planning methods,[8, 9] couples actively trying to conceive,[10-12] and couples undergoing artificial insemination where the female partner has normal reproductive function.[13,14] Differences between populations, including prior fertility history, age distribution, coital frequency, intent to conceive, and timing and method of conception confirmation contribute to variation in the estimates. All of these populations are likely to have higher fecundability then many potential clinical trial subjects with acute or chronic illness.

These estimates range from approximately 20% per cycle in nulliparous women under 30 undergoing donor insemination when only clinical pregnancies were the primary outcome[13] to 40% per cycle in Chinese textile workers aged 20-34 who were actively trying to conceive when daily urines were tested with a highly sensitive hCG assay.[12] For the purposes of this analysis, we used an estimate of **30%** per cycle for women 30 and younger, which is consistent with the observed rate in early cycles in a US population-based study,[15] and with a European study of couples using natural family planning methods who had a coital frequency of twice a week.[9] Although all prospective studies show a decline in cycle-specific probability as the number of cycles increases beyond 2 or 3 (representing heterogeneity in underlying fertility), we conservatively assumed that the cycle-specific probability remained constant over the duration of the simulation (which leads to an overestimation of cumulative pregnancy probability).

Figure 3: Cycle-specific Conception Probability by Age

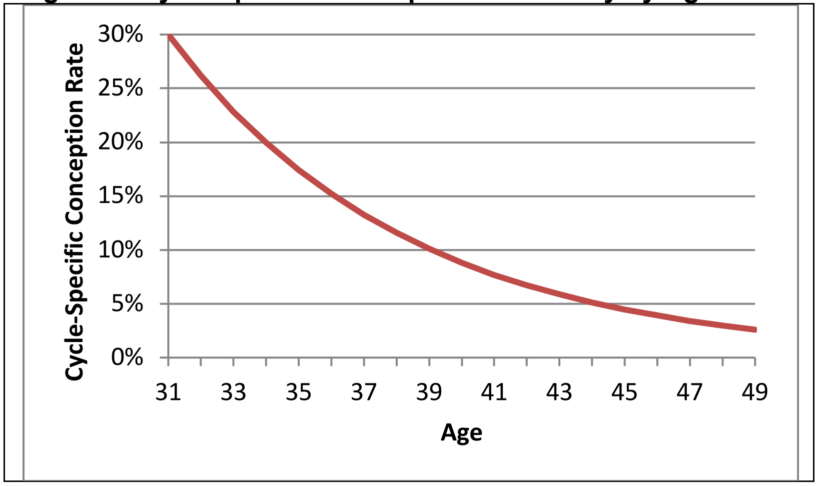

We modeled the effect of age using published regression equations based on a Dutch study of donor insemination[13]; in this study, a "critical age" (31 years) was identified after which there was a statistically significant progressive decline in fecundability. Although there is evidence that cycle fecundity begins to decrease prior to age 30,[9,16] we again elected to be conservative and assumed no decline in fecundity until age 32, when the hazard ratio derived from the van Noord-Zaadsra paper was applied (Figure 3).

F. Pregnancy Outcome Probabilities

Pregnancy loss after conception is common, with a substantial proportion occurring after implantation but before pregnancy is suspected clinically.[11,12,14,15] Studies using extremely sensitive assays report early pregnancy loss rates (prior to the onset of the next menses) of 22-23%,[12,15] while rates in two studies using less sensitive assays were 13%.[11,14] Confidence intervals for the 3 US-based studies overlapped; while the Chinese study [12] was large and population-based, the population was somewhat different in terms of potential exposures that might affect pregnancy loss (for example, prevalence of exposure to second-hand tobacco use in the home was 65%). There were also differences in the definition of "early pregnancy loss" between studies, ranging from detectable hCG with no delay in menses[11] or "clinical detection"[12,15] to losses prior to 23 days after the serum LH surge (9 days after the expected onset of menses).[14]

Because the sensitivity of the assay used in the Wilcox study was higher than those in the Lohstroh and Zinaman papers, and higher than current commercially available kits, and because 21% of the subjects with "non-clinical" pregnancy losses in the Wilcox study actually had pregnancy symptoms,[17] which lowers the proportion of truly non-clinical losses to 18%, we elected to use the lower estimates form Lohstroh and Zinaman. Because the proportions were so similar, we pooled the total pregnancies by

category for the two studies, and generated a Dirichlet distribution for the conditional probability of each pregnancy outcome, given conception (Table 4).

Table 4: Distribution of pregnancy outcomes, given conception

Outcome	N	Proportion (95% CI)
Live birth	114	13.4% (9.3-19.6%)
Clinical loss	35	20.2% (14.4-26.2%)
"Nonclinical" pregnancy	24	66.0% (58.0-72.9%)

Although clinical, and probably occult, pregnancy loss rates clearly increase with age,[18] we did not attempt to model the relative conditional probability of pregnancy loss with age. We also did not include ectopic pregnancy, gestational trophoblastic neoplasia, or other uncommon outcomes.

G. Contraceptive Method

For the initial analysis, we assumed that women would continue to use their current method of contraception. Although this is appropriate for estimating the prior probability of pregnancy at the time of the initial screen, protocols may specify methods that may be used during the protocol—the model can be readily adjusted to accommodate protocol-specific methods. We also assume that the population-based estimates of method choice would be similar to those in a patient population. While this may be true for some conditions, certain methods may be more or less prevalent in other conditions. For example, patients with acne may be more likely to be using oral contraceptives, given their effectiveness in reducing circulating androgen levels, while patients on some anti-epilepsy drugs might be less likely to be on oral contraceptives, given the potential for mutual effects on drug metabolism.

Estimates for the age-specific distribution of specific methods among women using contraception were derived from the 2006 National Survey of Family Growth (NSFG) (Table 5).[19] To minimize the number of categories for modeling, we combined methods with similar effectiveness where appropriate (oral contraceptives, contraceptive rings and patches as one category, IUDs and hormonal implants—long-acting reversible contraceptives, or LARC—as another). We assumed that women using periodic abstinence as their primary method would be instructed to use barrier methods for the duration of the trial.

Table 5: Age-specific distribution of contraceptive methods

Method	Age (years)					
	15-19	20-24	25-29	30-34	35-39	40-44
BTL	0.0%	2.5%	16.2%	31.4%	40.1%	52.3%
Vasectomy	0.0%	0.8%	3.6%	8.9%	17.6%	20.5%
OCP	60.7%	57.3%	41.6%	29.1%	21.4%	11.9%
LARC	5.6%	7.7%	9.0%	8.5%	6.7%	4.3%
DMPA	9.7%	5.4%	5.6%	2.4%	1.0%	1.2%
Barrier	24.0%	26.3%	24.0%	19.6%	13.2%	9.8%

Because the NSFG data is based on a weighted survey sample design, and published age-specific proportions and standard errors are based on all women, including women who do not use contraception, we could not directly estimate confidence intervals around the distribution of methods within age groups. To indirectly account for the uncertainty in these estimates, we estimated the number of actual subjects in each age category by fitting a beta distribution to the reported proportion and standard error of women using contraception in each age group, then estimating the number of

women using each method based on the reported overall proportion. These numbers were then used to generate a Dirichlet distribution.

H. Contraceptive Effectiveness

We used the most recent published estimates of first-year, typical use 12 month method-specific failure rates[20] to generate estimates of effectiveness for each method. We made several simplifying assumptions, all of which result in higher estimates of the probability of pregnancy:

- We used “typical-use” rather than “perfect use” estimates.
- We used first-year estimates; typically, failure rates are highest in the first year of use for any method, due to both “selection” (couples with higher baseline fertility are more likely to get pregnant early), variability in user adherence in user-dependent methods such as oral contraception or barrier methods, and, for women in their 30s and 40s, age-dependent declines in fertility.
- We assumed a constant risk across all 12 months; again, failure rates are highest in the first few months after beginning use.
- We assumed that the reported relative reduction in pregnancy risk associated with each method was solely due to method effectiveness, rather than differences in the age distribution or intensity of desire to avoid pregnancy among users.

To generate estimates of the cycle specific relative reduction in pregnancy probability associated with specific methods:

- We assumed that, during the 12 months used to estimate failure rates, there were 13 menstrual cycles with a mean duration of 28 days.
- Assuming a constant risk of pregnancy and constant cycle-specific effectiveness, we generated estimates of the cycle-specific hazard of pregnancy with each method that was consistent with the reported 12 month failure rate.
- Using the estimated cycle-specific probability for non-contraceptive users as the reference, we then estimated hazard ratios for each method (because of the way the data are reported, we could not generate confidence intervals around these estimates) (Table 6).

Table 6: Contraceptive effectiveness

Method	12 Month Failure Rate	Per Cycle Hazard	Hazard Ratio
None	85.00%	13.50%	1.0000
Oral contraceptives	9.00%	0.70%	0.0519
Injectables	6.00%	0.45%	0.0333
Barrier methods	18.00%	1.40%	0.1037
CuT IUD	0.80%	0.07%	0.0048
Progestin IUD	0.20%	0.02%	0.0011
Female sterilization	0.55%	0.05%	0.0033
Male sterilization	0.01%	0.01%	0.0008
Hormonal Implant	0.05%	0.00%	0.0003
Hormonal ring/patch	9.00%	0.70%	0.0519

We then applied these hazard ratios to the age-specific pregnancy probability described in Section II.E, assuming no differential effect of contraceptive methods on detectable early pregnancy loss.

I. hCG levels in non-pregnant women

We estimated lognormal distributions for values of hCG in IU/L in serum and urine in non-pregnant women, stratified by age, from the reported quartiles in the papers by Cole and Ladner[21] and Snyder et al [22] (Table 7).

Table 7: hCG (IU/L) levels in non-pregnancy cycles

Source	Mean (95% CI)	Maximum	Reference
≤ 40 years old			
Urine			
LH surge ± 3 days	0.32 (0.05,1.6)	9.3	21
Other days	0.34 (0.11,0.81)	4.0	21
Serum	1.67 (1.06,2.5)	4.6	22
41-55 years old			
Urine	0.44 (0.02,2.4)	3.6*	21
Serum	3.6 (2.6,4.8)	7.7	22

*99th percentile; reported maximum in paper < reported 99th percentile

J. hCG levels in pregnancy

We assumed that the first day that hCG would be detectable in serum or urine would be 9 days after the serum LH peak (8 days after the urine peak), which is consistent across all studies which used LH as the marker for ovulation.[7,14,23,24] We generated Dirichlet distributions for the day of first hCG detection for live birth, clinical loss, and nonclinical losses using the values reported in Lohstroh et al[14]; because the number of events is small, the confidence intervals are quite wide.

We assumed that hCG would not be detectable in urine prior to serum; we again used the data published by Lohstroh et al to generate a Dirichlet distribution for the probability of different lag times between appearance in serum and in urine.[14]

Figure 4: Mean log hCG (IU/L) after detection

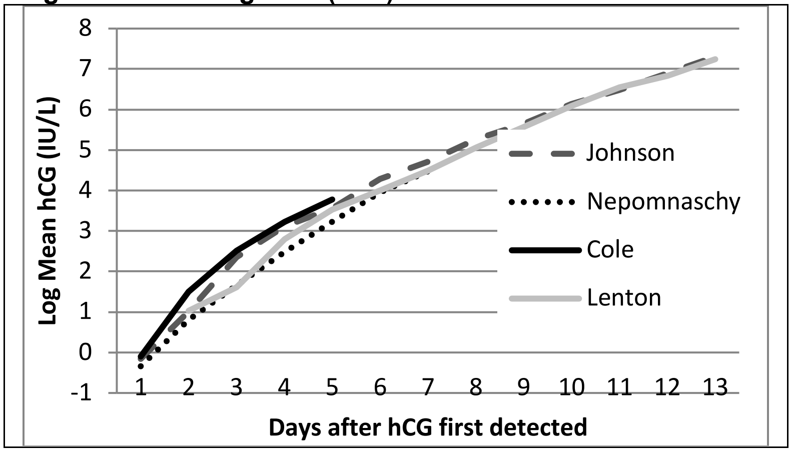

The rise in mean hCG after first day of detection is remarkably similar across studies (the values for Nepomnaschy et al[25] are converted to IU/L from ng/mL using the reported conversion factor), with values in urine[7,23,25] being indistinguishable from those in serum[24] (Figure 4). Because of the similarity across studies, we used the values (expressed as lognormal distributions) from Johnson et al,[23] which reflect currently available assays. We assumed that the pattern of increase would be similar in serum and urine; in the event of a delay between the first detectable serum value and first urine value, we assumed the curves would be similar, but would start from the same level (i.e., the curves would be in parallel, with a difference of 1-3 days.

We assumed that patterns of hCG increase in ongoing pregnancies, and falls in losses, would follow reported regression equations.[26,27]

K. Sensitivity of hCG assays

We did not attempt to quantify the analytic sensitivity of specific assays, or to quantify the impact of differential sensitivity for specific isoforms of hCG. For the purposes of comparing the probability of specific test outcomes (true and false negatives, true and false positives), we used the following values for analytic sensitivity:

- Serum hCG: 5 IU/L; because many labs report values between 5-14 or 5-24 as “indeterminate,” we used an alternative values of 15 and 25.
- Urine hCG: 20 IU/L, 25 IU/L, 50 IU/L.

L. Probability of pregnancy detection in the absence of testing

We used reported cumulative probability of reporting symptoms of pregnancy from a prospective study of couples attempting to conceive[17] to estimate the daily probability of a clinically detected pregnancy in the absence of testing. Based on the reported median and interquartile ranges using the timing of the LH surge as the reference, we first estimated the cumulative probability for women with live births using a Weibull function, and then calculated the day-to-day conditional probability from this curve. Estimates for women with clinical and the 21% of women with “non-clinical” losses with symptoms were generated using the reported day-to-day relative risks applied to the cumulative probabilities, then calculating day-specific conditional probabilities. Figure 5 depicts the derived cumulative probabilities for each group.

Figure 5: Estimated cumulative probability of pregnancy symptoms relative to urinary LH peak

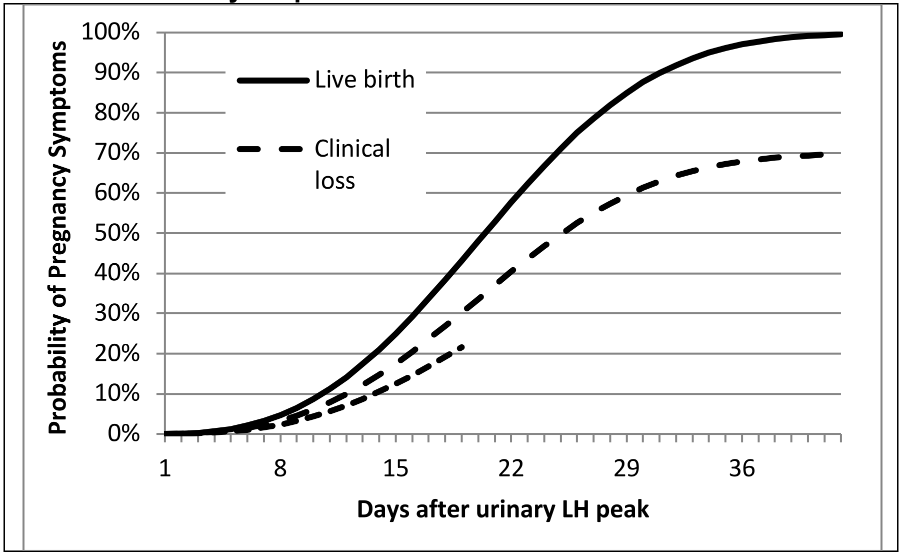

References

1. Strauss JS, Gottlieb AB, Jones T, Koo JY, Leyden JJ, Lucky A, et al. Concomitant administration of vitamin E does not change the side effects of isotretinoin as used in acne vulgaris: A randomized trial. J Am Acad Dermatol. 2000; 43(5, Part 1):777–84. doi: 10.1067/mjd.2000.110391. PMID: 11050580

2. French JA, Abou-Khalil BW, Leroy RF, Yacubian EM, Shin P, Hall S, et al. Randomized, double-blind, placebo-controlled trial of ezogabine (retigabine) in partial epilepsy. Neurology. 2011; 76(18):1555–63. doi: 10.1212/WNL.0b013e3182194bd3. PMID: 21451152

3. Simon GE, Ludman EJ, Tutty S, Operskalski B, Von Korff M. Telephone psychotherapy and telephone care management for primary care patients starting antidepressant treatment: A randomized controlled trial. JAMA. 2004; 292(8):935–42. doi: 10.1001/jama.292.8.935. PMID: 15328325

4. Breedveld FC, Weisman MH, Kavanaugh AF, Cohen SB, Pavelka K, van Vollenhoven R, et al. The PREMIER study: A multicenter, randomized, double-blind clinical trial of combination therapy with adalimumab plus methotrexate versus methotrexate alone or adalimumab alone in patients with early, aggressive rheumatoid arthritis who had not had previous methotrexate treatment. Arthritis Rheum. 2006;54(1):26–37. doi: 10.1002/art.21519. PMID: 16385520

5. Behavioral Risk Factor Surveillance System Survey Data. US Dept of Health and Human Services; 2010. Available at: http://apps.nccd.cdc.gov/s_broker/weatsql.exe/weat/index.hsql.

6. Gold EB, Bromberger J, Crawford S, Samuel S, Greendale GA, Harlow SD, et al. Factors associated with age at natural menopause in a multiethnic sample of midlife women. Am J Epidemiol. 2001; 153(9):865–74. PMID: 11323317

7. Cole LA, Ladner DG, Byrn FW. The normal variabilities of the menstrual cycle. Fertil Steril. 2009;91(2):522–7. doi: 10.1016/j.fertnstert.2007.11.073. PMID: 18433748

8. Colombo B, Masarotto G. Daily fecundability: first results from a new data base. Demogr Res. 2000; 3:[39] p.. PMID: 12178154

9. Dunson DB, Baird DD, Colombo B. Increased infertility with age in men and women. Obstet Gynecol. 2004; 103(1):51–6. doi: 10.1097/01.AOG.0000100153.24061.45. PMID: 14704244

10. Wilcox AJ, Weinberg CR, Baird DD. Timing of sexual intercourse in relation to ovulation. Effects on the probability of conception, survival of the pregnancy, and sex of the baby. N Engl J Med. 1995; 333(23):1517–21. doi: 10.1056/NEJM199512073332301. PMID: 7477165

11. Zinaman MJ, Clegg ED, Brown CC, O'Connor J, Selevan SG. Estimates of human fertility and pregnancy loss. Fertil Steril. 1996; 65(3):503–9. PMID: 8774277

12. Wang X, Chen C, Wang L, Chen D, Guang W, French J. Conception, early pregnancy loss, and time to clinical pregnancy: a population-based prospective study. Fertil Steril. 2003; 79(3):577–84. PMID: 12620443

13. van Noord-Zaadstra BM, Looman CW, Alsbach H, Habbema JD, te Velde ER, Karbaat J. Delaying childbearing: effect of age on fecundity and outcome of pregnancy. BMJ. 1991; 302(6789):1361–5. PMID: 2059713

14. Lohstroh PN, Overstreet JW, Stewart DR, Nakajima ST, Cragun JR, Boyers SP, Lasley BL. Secretion and excretion of human chorionic gonadotropin during early pregnancy. Fertil Steril. 2005;83(4):1000–11. doi: 10.1016/j.fertnstert.2004.10.038. PMID: 15820813

15. Wilcox AJ, Weinberg CR, O'Connor JF, Baird DD, Schlatterer JP, Canfield RE, et al. Incidence of early loss of pregnancy. N Engl J Med. 1988; 319(4):189–94. doi: 15820813

16. Dunson DB, Colombo B, Baird DD. Changes with age in the level and duration of fertility in the menstrual cycle. Hum Reprod. 2002; 17(5):1399–403. PMID: 1198077

17. Sayle AE, Wilcox AJ, Weinberg CR, Baird DD. A prospective study of the onset of symptoms of pregnancy. J Clin Epidemiol. 2002; 55(7):676-680. PMID: 1216091

18. Nybo Andersen AM, Wohlfahrt J, Christens P, Olsen J, Melbye M. Maternal age and fetal loss: population based register linkage study. BMJ. 2000; 320(7251):1708–12. PMID: 10864550

19. Mosher WD, Jones J. Use of contraception in the United States: 1982-2008. Vital Health Stat 23. 2010; Aug(29):1–44. PMID: 20939159

20. Trussell J. Contraceptive failure in the United States. Contraception. 2011; 83(5):397–404. doi: 10.1016/j.contraception.2011.01.021. PMID: 21477680

21. Cole LA, Ladner DG. Background hCG in non-pregnant individuals: Need for more sensitive point-of-care and over-the-counter pregnancy tests. Clin Biochem. 2009; 42(3):168–75. doi: 10.1016/j.clinbiochem.2008.09.107. PMID: 18929550

22. Snyder JA, Haymond S, Parvin CA, Gronowski AM, Grenache DG. Diagnostic considerations in the measurement of human chorionic gonadotropin in aging women. Clin Chem. 2005; 51(10):1830–5. doi: 10.1373/clinchem.2005.053595. PMID: 16099935

23. Johnson SR, Miro F, Barrett S, Ellis JE. Levels of urinary human chorionic gonadotrophin (hCG) following conception and variability of menstrual cycle length in a cohort of women attempting to conceive. Curr Med Res Opin. 2009; 25(3):741–8. doi: 10.1185/03007990902743935. PMID: 19196217

24. Lenton EA, Neal LM, Sulaiman R. Plasma concentrations of human chorionic gonadotropin from the time of implantation until the second week of pregnancy. Fertil Steril. 1982; 37(6):773–8. PMID: 7115557

25. Nepomnaschy PA, Weinberg CR, Wilcox AJ, Baird DD. Urinary hCG patterns during the week following implantation. Hum Reprod. 2008; 23(2):271–7. doi: 10.1093/humrep/dem397. PMID: 18083748

26. Barnhart KT, Sammel MD, Rinaudo PF, Zhou L, Hummel AC, Guo W. Symptomatic patients with an early viable intrauterine pregnancy: hCG curves redefined. Obstet Gynecol. 2004; 104(1):50–5. doi: 10.1097/1001.AOG.0000128174.0000148843.0000128112. PMID: 15229000

27. Barnhart K, Sammel MD, Chung K, Zhou L, Hummel AC, Guo W. Decline of serum human chorionic gonadotropin and spontaneous complete abortion: defining the normal curve. Obstet Gynecol. 2004; 104(5 Pt 1):975–81. doi: 10.1097/01.AOG.0000142712.80407.fd. PMID: 15516387
